# Intensive blood pressure lowering for ischemic stroke patients: does it prevent ischemia or bleeding?

**DOI:** 10.1038/s41440-022-00892-6

**Published:** 2022-03-22

**Authors:** Kazunori Toyoda

**Affiliations:** Department of Cerebrovascular Medicine, National Cerebral and Cardiovascular Center, Suita, Osaka, Japan

**Fig. 1:**
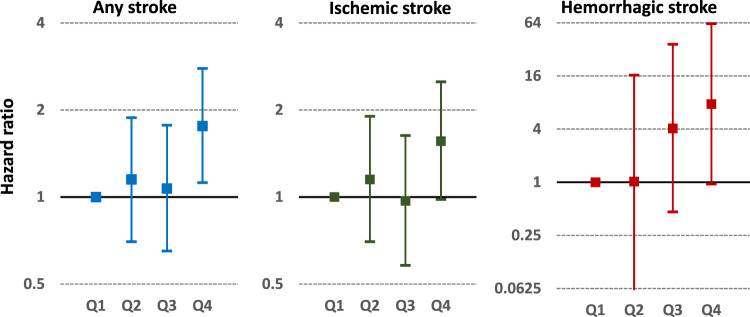
The risk of incident stroke by mean systolic blood pressure quartiles in the PRASTRO-1 trial. A total of 3747 patients with noncardioembolic ischemic stroke randomly assigned (1:1) to receive prasugrel or clopidogrel are divided into quartiles according to mean follow-up systolic blood pressure levels (Q1: ≤ 126.1 mmHg, Q2: 126.1–132.5 mmHg, Q3: 132.5–138.6 mmHg, Q4: > 138.6 mmHg). The risks of any, ischemic, and hemorrhagic strokes by quartiles during the median follow-up of 1.8 years are shown. The markers represent the hazard ratios relative to Q1. The bars show the 95% confidence intervals. Logarithmic scales are used for the *y*-axes. Edited based on data of Ref. [3]

The Perindopril Protection Against Recurrent Stroke Study (PROGRESS) showed the benefits of blood pressure (BP) lowering for secondary stroke prevention in stroke survivors for the first time after a long dispute in the last century [1] and triggered active hypertensive therapy for stroke patients globally in this century. A post hoc analysis of PROGRESS demonstrated that both the lowest risks of ischemic stroke and of hemorrhagic stroke were among the one-quarter of patients with the lowest achieved follow-up BP levels (median 112/72 mmHg), and that these risks increased progressively with higher follow-up BP levels in the large cohort of patients with previous stroke or transient ischemic attack [2]. A similar tendency for an increased risk of ischemic/hemorrhagic stroke by increased follow-up BP levels was reproduced in subsequent stroke trials (Fig. 1) [3]. Several trials sought to clarify the preventive effect against recurrent stroke by randomizing patients into two groups with different BP lowering targets. In patients with recent lacunar stroke in the Secondary Prevention of Small Subcortical Strokes (SPS3) trial, lowering systolic BP to <130 mmHg significantly reduced the risk of intracerebral hemorrhage (ICH) by 63% and insignificantly reduced the risk of ischemic stroke by 16% compared to lowering to 130–149 mmHg [4]. The Recurrent Stroke Prevention Clinical Outcome (RESPECT) Study, involving patients having a history of stroke within the previous 3 years showed similar results: lowering BP to <120/80 mmHg, relative to <140/90 mmHg, reduced the risk of ICH by 91% and that of ischemic stroke by only 9% [5]. These trials failed to demonstrate a significant reduction in the risk of any stroke as the primary outcome by intensive BP lowering, since the incidence of ischemic stroke was much higher than that of ICH for both trials. A meta-analysis of these two trials and two more small studies finally succeeded in showing a statistically significant 22% reduced risk for any stroke recurrence by lowering systolic BP to at least <130 mmHg [5].

In the present post hoc analysis of RESPECT, Kitagawa et al. examined the effect of intensive BP lowering on recurrent stroke subtype risk in patients with a history of ischemic stroke, accounting for 84% of the overall participants in RESPECT [6]. The major finding was that strict BP control aiming at <120/80 mmHg significantly reduced the risk of ICH. Patients assigned to intensive BP lowering did not develop ICH for a mean 3.9-year follow-up, whereas 0.39% of patients assigned to standard BP lowering developed ICH annually. It is less likely that the J-curve phenomenon exists between the follow-up BP levels and the risk of ICH relative to that of ischemic stroke, since possible cerebral hypoperfusion due to systemic hypotension rarely causes bleeding events. Most patients developing ischemic stroke took antithrombotic agents like the present RESPECT cohort, and higher BP levels during antithrombotic medication were associated with incidental ICH, as previous studies on secondary stroke prevention with antithrombotic therapy indicated [3, 4, 7]. A limitation of the analysis of the risk of ICH common to RESPECT and other previous studies is that the incidence of ICH was too low for detailed investigation.

Another major finding of the RESPECT sub-analysis was that the risk of recurrent ischemic stroke was almost identical between two treatment groups with different BP lowering targets [6]. The unclear preventive effect against ischemic stroke relative to ICH by intensive BP lowering was similar to the main results of RESPECT and other published studies. Based on the present result, the lower goal of <120/80 mmHg compared to previous trials does not seem to be necessary for secondary prevention of ischemic stroke in patients with a history of ischemic stroke. However, there are several points of discussion regarding this second major finding. First, we usually assume that cerebral hypoperfusion is the mechanism of ischemic stroke for patients with a very low BP level. However, the risk of atherothrombotic infarction halved and that of lacunar infarction increased somewhat by intensive BP lowering in this sub-analysis, opposite to our expectation. Ischemic stroke subtypes as outcome events are influenced by the subtype of qualifying stroke, which was not clarified in this article. Second, the neurological severity or disability after ischemic stroke was not documented. Patients developing atherothrombotic infarction and cardioembolism were relatively few, and those with lacunar stroke and transient ischemic attack were relatively frequent in the intensive treatment group. Thus, intensive BP lowering might be beneficial for preventing disabling ischemic stroke. In any case, the number of each incidental stroke subtype was too small for further analysis to respond to the first and second discussion points. Third, mean baseline systolic BP of this sub-analysis cohort was ≈140 mmHg, lower than previous studies. In PROGRESS, the risk of hemorrhagic stroke was lower in the active hypertensive treatment group than in the placebo group regardless of baseline BP levels, but the risk of ischemic stroke did not decrease significantly with active treatment in the sub-groups with lower baseline BP levels [2]. Fourth, the mean follow-up BP was 126.7/74.1 mmHg in the intensive treatment group and 133.4/77.5 mmHg in the standard treatment group, and the average difference in systolic BP of 6.7 mmHg was much smaller than the planned one of ≈20 mmHg. Such a small difference would make the therapeutic effect of intensive BP lowering unclear.

As the adverse events of intensive BP lowering, the authors of the RESPECT sub-analysis indicated caution regarding falls and renal dysfunction [6]. Acute kidney injury is a known complication of intensive BP lowering during acute stroke [8, 9], but similar ischemic damage to the kidneys would also be a concern in chronic stroke patients. Table 1 shows factors favoring strict and those favoring gentle antihypertensive therapy for ischemic stroke survivors based on the findings of PROGRESS and other studies. Relatively low mortality in the intensive treatment group, although statistically insignificant, was another interesting finding.

**Table 1:** Factors favoring strict and those favoring gentle antihypertensive therapy for ischemic stroke survivors

Factors favoring strict antihypertensive therapy	Factors favoring gentle antihypertensive therapy
• High bleeding risk • Antithrombotic medication • Cerebral microbleeds • Co-existent cardiovascular disease: coronary artery disease, heart failure, aortic aneurysm, etc.	• Steno-occlusive disease of cervical and intracranial arteries related to cerebral hemodynamic failure • Risk of renal ischemia • Falls, orthostatic dysregulation

The current guidelines recommend systolic BP control aiming at <130 mmHg or 120–130 mmHg for adults who experience ischemic stroke [10–12]. RESPECT is epochal as the first large population trial to examine the importance of systolic BP lowering to <120 mmHg. Such strict control would undoubtedly decrease incidental ICH, especially for patients taking antithrombotic agents. However, the necessity of strict BP control for protection of brain tissues from ischemic injury is still an unresolved matter that needs to be addressed in future studies.
